# Incidence of airway complications in patients using endotracheal tubes with continuous aspiration of subglottic secretions

**DOI:** 10.1186/s13613-017-0331-0

**Published:** 2017-11-02

**Authors:** Jordi Vallés, Susana Millán, Emili Díaz, Eva Castanyer, Xavier Gallardo, Ignacio Martín-Loeches, Marta Andreu, Mario Prenafeta, Paula Saludes, Jorge Lema, Montse Batlle, Néstor Bacelar, Antoni Artigas

**Affiliations:** 1 Critical Care Department, Hospital-Sabadell, Corporació Sanitària Universitària Parc Taulí, ParcTauli s/n, 08208 Sabadell, Spain; 2 Universitat Autonoma Barcelona, Sabadell, Spain; 3 CIBERES Enfermedades Respiratorias, Valladolid, Spain; 4 UDIAT, Hospital Universitari Parc Taulí, Sabadell, Spain

**Keywords:** Mechanical ventilation, Ventilator-associated pneumonia, Continuous aspiration of subglottic secretions

## Abstract

**Background:**

Continuous aspiration of subglottic secretions is effective in preventing ventilator-associated pneumonia, but it involves a risk of mucosal damage. The main objective of our study was to determine the incidence of airway complications related to continuous aspiration of subglottic secretions.

**Methods:**

In consecutive adult patients with continuous aspiration of subglottic secretions, we prospectively recorded clinical airway complications during the period after extubation. A multidetector computed tomography of the neck was performed during the period of 5 days following extubation to classify subglottic and tracheal lesions as mucosal thickening, cartilage thickening or deep ulceration.

**Results:**

In the 86 patients included in the study, 6 (6.9%) had transient dyspnea, 7 (8.1%) had upper airway obstruction and 18 (20.9%) had dysphonia at extubation. Univariate analysis identified more attempts required for intubation (2.3 ± 1.1 vs. 1.2 ± 0.5; *p* = 0.001), difficult intubation (71.4 vs. 10.1%, *p* = 0.001) and Cormack score III–IV (71.4 vs. 8.8%; *p* < 0.001) as risk factors for having an upper airway obstruction at extubation. The incidence of failed extubation among patients after planned extubation was 18.9% and 11 patients (12.7%) required tracheostomy. A multidetector computed tomography was performed in 37 patients following extubation, and injuries were observed in 9 patients (24.3%) and classified as tracheal injuries in 2 patients (1 cartilage thickening and 1 mild stenosis with cartilage thickening) and as subglottic mucosal thickenings in 7 patients.

**Conclusions:**

The incidence of upper airway obstruction after extubation in patients with continuous aspiration of subglottic secretions was 8.1%, and the injuries observed by computed tomography were not severe and located mostly in subglottic space.

## Background

More than 500,000 patients with acute respiratory failure receive invasive mechanical ventilation in the USA each year [1, 2]. The most frequent complications that develop during mechanical ventilation are ventilator-associated infections, especially ventilator-associated pneumonia (VAP). VAP is associated with increased morbidity and mortality [3, 4].

VAP is mainly due to the use of an artificial airway and repeated microaspirations of secretions from the oropharynx containing microorganisms through the space between the endotracheal tube and the tracheal wall.

In recent decades, multiple pharmacological and non-pharmacological prevention strategies administered individually or in bundles have lowered the incidence of VAP [5].

One recommended non-pharmacological measure reduces microaspirations by using an endotracheal tube with an accessory channel that allows subglottic secretions accumulated above the endotracheal cuff to be removed. These tubes allow secretions to be aspirated intermittently or continuously [6–8].

Although continuous aspiration of subglottic secretions (CASS) is effective in preventing VAP, it involves a risk of mucosal damage secondary to aspiration because the external diameter is higher than conventional tubes. One experimental study [9] and some clinical reports suggest that CASS can cause subglottic injuries around the point in the endotracheal tube where secretions are suctioned with the possibility of secondary laryngeal edema and upper airway obstruction [10–12]. However, clinical studies focusing on subglottic and tracheal damage associated with CASS are lacking.

Thus, we aimed to analyze the incidence of significant clinical complications and tracheal damage level in patients intubated with an endotracheal tube with CASS as a method to prevent VAP.

## Methods

### Study population

This was a prospective observational study of patients admitted to the intensive care unit (ICU) of a university hospital (between December 1, 2013, and November 30, 2014).

All patients aged > 18 years intubated with endotracheal tubes with CASS and who were expected to require mechanical ventilation for at least 48 h were eligible. In our hospital, all patients admitted to the ICU who require mechanical ventilation are intubated with endotracheal tubes that allow subglottic aspiration. We excluded patients transferred from hospitals who were intubated with endotracheal tubes without accessory channel that allows subglottic aspiration, those with a history of tracheostomy or tracheal lesions (including laryngeal surgery), those intubated > 48 h in the 30 days prior to the current admission and those enrolled in other trials.

Tracheal tube size was 7.5 and 8 in women and men, respectively, as used in the study of Touat et al. [13] with conventional tubes. All patients included in the study were intubated with tracheal tubes with an accessory channel for subglottic aspiration (Mallinckrodt™ TaperGuard™ Evac; Covidien Healthcare; Mansfield, MA, USA). The aspiration pressure was continuously monitored and maintained at 20 mmHg with a continuous vacuum regulator (Push-To-Set™;Ohio Medical; Gurnee, IL, USA). By protocol in our ICU, cuff pressure is checked intermittently every 4 h with a hand pressure gauge (Mallinckrodt™; Covidien Healthcare; Mansfield, MA, USA) to maintain it between 20 cm H_2_O and 30 cm H_2_O. Systemic corticosteroids were not used to prevent post-extubation laryngeal edema.

### Data collection

The following data were prospectively recorded at ICU admission: age, sex, Acute Physiology and Chronic Health Evaluation II (APACHE II) score at admission, reason for ICU admission, comorbidities (diabetes, chronic obstructive pulmonary disease (COPD), chronic liver failure, chronic heart failure, chronic kidney disease requiring dialysis, cancer, previous surgery and immunosuppression) and information related to tracheal intubation (date, whether urgent or scheduled, place (outpatient or inpatient), size of tracheal tube, Cormack–Lehane score, number of attempts and need for auxiliary devices, such as laryngeal mask or video laryngoscopy. We defined a difficult airway as the clinical situation in which a conventionally trained physician experiences difficulty with facemask ventilation of the upper airway, difficulty with tracheal intubation or both [14]; specific criteria for difficult intubation were Cormack–Lehane score grade III or IV, more than two attempts and need for auxiliary devices for intubation.

The following data were recorded during the ICU stay: self-extubations, reintubations and cause of reintubation, tracheostomy, mean daily aspiration pressure of the subglottic space, post-extubation complications (upper airway obstruction, dysphagia, dyspnea or dysphonia), days intubated, VAP, length of ICU stay and ICU mortality. Upper airway obstruction was considered when the patient presented stridor secondary to laryngeal edema after the extubation. The patients with planned extubation were extubated following a spontaneous breathing trial with a standard test for extubation using the T-piece for 30 min.

### Diagnosis of tracheal lesions

To detect damage of the larynx and trachea, patients’ airways were studied within 5 days of extubation using a 128-row multidetector computed tomography (MDCT) scanner (Somaton Definition AS plus; Siemens Healthcare; Erlangen, Germany) which obtained 1 mm slices with a 0.75-mm interslice gap from the vocal cords to 2 cm below the carina focused on the airway. No MDCT studies were carried out in patients aged < 50 years or pregnant women (to avoid possible adverse effects secondary to radiation), in patients with tracheostomies or previously known tracheal lesions (including laryngeal surgery), in patients mechanically ventilated < 72 h or in terminal patients extubated in order to withdraw life support or in patients without consent.

Four independent radiologists blinded to clinical information analyzed the MDCT images and classified the site (the arytenoid region, the subglottic region or the trachea to 2 cm below the carina) and severity of injuries (from mildest to most severe: mucosal thickening, cartilage thickening or deep ulceration) and evaluated whether tracheal stenosis was present. The group reached a consensus on discrepant readings.

### Statistical analysis

Categorical variables are reported as frequencies (%), and normally distributed continuous variables are reported as mean ± SD. We used the Chi-square test or Fischer’s exact test to compare qualitative variables and Student’s *t* test or the Mann–Whitney *U* test to compare continuous variables. All tests were two-tailed, and significance was set at *p* < 0.05. SPSS software (SPSS Inc.; Chicago, IL, USA) was used for data all analysis.

## Results

**Fig. 1:**
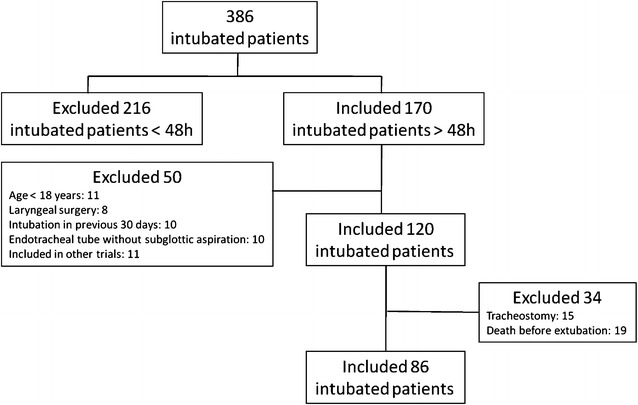
Study design and patients included

During the study period, 386 patients admitted to the ICU were mechanically ventilated. Of these, 266 were excluded: 216 because they were ventilated < 48 h and 50 for other reasons (Fig. 1). Thus, a total of 120 patients were enrolled in the study; however, 34 of these were excluded: 19 because they died before extubation and 15 because they were tracheostomized before extubation was attempted.

The study population consisted of the remaining 86 patients (mean age, 61 ± 14 years; 55 (64%) male; mean Apache II at admission, 18 ± 2). Table 1 reports the characteristics of these patients. Table 2 reports the variables recorded during the intubation procedure and the ICU stay. Nearly all patients (95.4%) were intubated inside the hospital, and most intubations (89.5%) were urgent. A total of 15.1% of patients fulfilled the criteria of difficult intubation. The incidence rate of VAP during the study period was 4.7 episodes per 1000 days of mechanical ventilation. ICU mortality of 120 patients initially included was 21.6%, and the mortality of 86 extubated patients was 8.1%.

**Table 1:** Patient characteristics at ICU admission

Characteristics	*N*:86
Age, mean ± SD	61 ± 14
Male (%)	55 (64)
Apache II, mean ± SD	18 ± 2
Reason for admission (%)	
Acute respiratory failure	22 (25.5)
Neurological illness	18 (21.0)
Severe sepsis or septic shock	14 (16.3)
Shock	11 (12.7)
Other^a^	21 (24.4)
Comorbidities (%)	
Diabetes	24 (28.0)
Cardiovascular	21 (24.4)
COPD	17 (19.7)
Cancer	17 (19.7)
Immunosuppression	13 (15.0)
Chronic liver failure	8 (9.3)
Chronic renal failure	5 (5.8)

*COPD* chronic obstructive pulmonary disease

^a^Intoxications, trauma, cardiac arrest, gastrointestinal bleeding

**Table 2:** Patient characteristics during intubation and during ICU stay

Characteristics	*N*:86
During intubation procedure	
Number of attempts required for intubation, mean ± SD	1.3 ± 0.6
Diameter of endotracheal tube (mm), mean ± SD	7.8 ± 0.3
Difficult intubation (%)	13 (15)
Urgent intubation (%)	77 (89.5)
Intubation outside the hospital (%)	4 (4.6)
During ICU stay	
Subglottic aspiration pressure (mmHg), mean ± SD	19.7 ± 2.5
Length of intubation (days), mean ± SD	6.1 ± 4.8
Accidental or self-extubation (%)	7 (8.1)
Planned extubation (%)	79 (91.8)
Reintubation (%)	18 (20.9)
Tracheostomy (%)	11 (12.7)
Post-extubation complications (%)	
Dyspnea	6 (6.9)
Upper airway obstruction (Stridor)	7 (8.1)
Severe dysphagia > 24 h^a^	0
Dysphonia	18 (20.9)
Length of ICU stay (days), mean ± SD	16.4 ± 23.3
Crude mortality (%)	7 (8.1)

^a^Excluded patients with tracheostomy or with neurological disorders

**Fig. 2:**
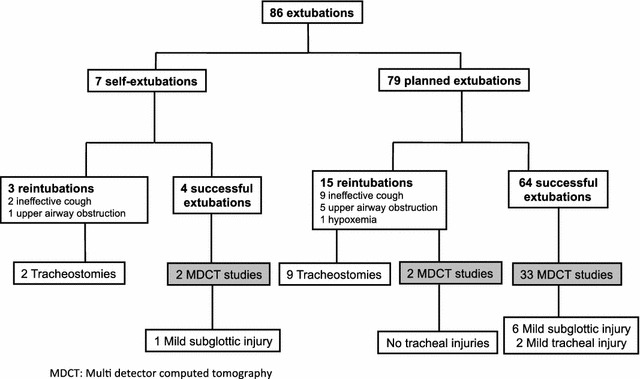
Post-extubation complications and results of MDCT studies

Of the 86 extubated patients, 79 (91.8%) underwent a planned extubation and 7 (8.1%) had accidental or self-extubation (Fig. 2). Among patients with accidental or self-extubation, 3 (42.8%) required reintubation (2 for ineffective cough with airway secretion buildup and 1 for upper airway obstruction), and 2 of these required tracheostomy. Among patients extubated after planned extubation, 15 (18.9%) required reintubation (9 for ineffective cough with airway secretion buildup, 5 for upper airway obstruction and 1 for hypoxemia), and 9 of these required tracheostomy. Among the 15 patients extubated after planned extubation who needed reintubation, 7 (46.6%) were neurocritical patients. Compared with patients without reintubation, ICU mortality in patients who required reintubation was 33.3 versus 1.5% (*p* < 0.001).

Post-extubation complications included dysphonia in 18 (20.9%) patients, upper airway obstruction in 7 (8.1%) patients and transient dyspnea in 6 (6.9%). Excepting patients with tracheostomy or neurological disorders, none of the extubated patients had severe dysphagia impeding swallowing > 24 h after extubation. Univariate analysis showed that the significant risk factors for upper airway obstruction were higher attempts for intubation, the presence of difficult intubation and a higher Cormack score (Table 3).

**Table 3:** Characteristics of patients with upper airway obstruction

Characteristics	Upper airway obstruction (*N*:7)	Non-upper airway obstruction (*N*:79)	*p* value
Length of intubation (days), mean ± SD	5.9 ± 2.8	6.0 ± 5.0	0.90
Attempts at intubation, mean ± SD	2.3 ± 1.1	1.2 ± 0.5	0.001
Cormack–Lehane score II–IV (%)	5 (71.4)	7 (8.8)	< 0.001
Difficult intubation (%)	5 (71.4)	8 (10.1)	0.001
Intubation outside the hospital (%)	0	4 (5.1)	0.70
Crude mortality (%)	1 (14.3)	6 (7.6)	0.53

Thirty-seven patients (43.02%) underwent MDCT study. The remaining 49 patients were excluded by protocol. (Sixteen were intubated < 72 h, 12 were aged < 50 years, 11 were tracheostomized, 6 did not consent and 4 were extubated to withdraw life support.) The mean time between the MDCT study and extubation was 5 ± 2 days. There were no significant differences between patients who underwent MDCT and extubated patients who did not. Mean age and APACHE II were higher in those undergoing MDCT because only patients > 50 years we included (Table 4).

**Table 4:** Characteristics of patients included in the multidetector computed tomography study

Characteristics	MDCT (*N*:37)	No MDCT (*N*:49)	*p* value
Age, mean ± SD	64.7 ± 8.9	58.7 ± 16.2	0.04
Apache II, mean ± SD	18.8 ± 1.6	17.7 ± 1.9	0.005
Airway and intubation			
Cormack–Lehane score III–IV (%)	5 (13.5)	7 (14.2)	0.91
Number of attempts required for intubation, mean ± SD	1.3 ± 0.6	1.3 ± 0.4	0.40
Diameter of endotracheal tube (mm), mean ± SD	7.8 ± 0.2	7.8 ± 0.3	0.49
Subglottic aspiration pressure (mmHg), mean ± SD	19.4 ± 3.4	19.9 ± 1.6	0.34
Difficult intubation (%)	6 (16.2)	7 (14.3)	0.80
Urgent intubation (%)	34 (91.8)	43 (87.8)	0.53
Stridor (%)	2 (5.4)	5 (10.2)	0.69
Dysphonia (%)	7 (18.9)	9 (18.3)	0.77
Length of ICU stay (days), mean ± SD	6.5 ± 3.9	5.8 ± 5.6	0.67

*MDCT* multidetector computed tomography

MDCT showed lesions in 9 out of 37 patients (24.3%), 2 had tracheal lesions (1 cartilage thickening and 1 mild stenosis and cartilage thickening) and 7 had mucosal thickening in the subglottic space. There were no significant differences between patients with lesions on the MDCT and those without, except mean subglottic aspiration pressure was unexpectedly higher in the group without lesions (*p* = 0.002) (Table 5).

**Table 5:** Characteristics of patients with injuries detected at multidetector computed tomography

Characteristics	Airway injury (*N*:9)	No airway injury (*N*:28)	*p* value
Age, mean ± SD	62.4 ± 10.8	65.5 ± 8.2	0.20
Apache II, mean ± SD	19 ± 1	19 ± 2	0.08
Airway and intubation			
Cormack–Lehane score III–IV (%)	1 (11.1)	4 (14.2)	1.00
Number of attempts required for intubation, mean ± SD	1.4 ± 0.8	1.4 ± 0.7	0.60
Diameter of endotracheal tube (mm), mean ± SD	7.7 ± 0.3	7.8 ± 0.2	0.12
Subglottic aspiration pressure (mmHg), mean ± SD	17.8 ± 6.6	19.9 ± 1	0.002
Difficult intubation (%)	2 (22)	4 (14)	0.62
Urgent intubation (%)	9 (100)	25 (89.3)	0.56
Length of ICU stay (days), mean ± SD	7.4 ± 3.7	6.2 ± 2.0	0.80

## Discussion

The incidence of clinically significant tracheal lesions in our patients using CASS was not higher than that reported in intubated patients without CASS, and suggests that CASS to prevent VAP is safe, at least when the pressure of subglottic aspiration is maintained at a safe level (20 mmHg).

MDCT was performed in 43% of extubated patients and found structural subglottic or tracheal injuries in 23.4% of them. However, MDCT could have underdiagnosed the incidence of lesions compared with fiber optic tracheoscopy. Touat et al. [13] recently reported that tracheoscopy within 24 h after extubation found at least one ischemic lesion in the cuff contact area in 83% of patients. Most lesions were mild and classified as edema or hyperemia. In the early 1980s, a study using fiber optic tracheoscopy found an incidence of ischemic lesions in 31% [15], and another using postmortem analysis found an incidence of 95% [16].

However, several studies have demonstrated the usefulness of MDCT in the detection of moderate and severe subglottic and tracheal lesions (thickenings, ulcers, granulomas, stenosis), with a 90% sensitivity compared to fiber optic tracheoscopy studies [17–20]. Although MDCT probably detected the most severe injuries, it probably failed to detect clinically insignificant injuries such as those reported in Touat et al.’s [14] study, where most lesions were mild and described as edema or hyperemia.

In their experimental study in sheep, Berra et al. [9] studied the tracheal injuries caused by CASS and classified most injuries as erythemic or hemorrhagic. The incidence of more severe lesions such as necrosis and cartilage thickening was less common. The objective of our study was to detect severe injuries related to CASS. Although MDCT is able to detect the majority of these severe injuries, the sensitivity of MDCT to detect mild injuries without significant clinical repercussion may be less than in tracheoscopy studies.

In our study, the group with injuries had a significantly lower mean subglottic aspiration pressure than in the group without lesions. This finding was unexpected because our hypothesis stated that more injuries should have occurred with a higher subglottic aspiration pressure. However, it is possible that the level of subglottic aspiration is not directly related to the tracheal injuries if the level of aspiration is maintained < 20 mmHg. On the other hand, other variables such as the difficult or urgent intubation were higher in the group of patients with injuries detected by MDCT and could therefore be responsible for the lesions. However, these differences were not statistically significant which could be due to the small number of patients included in the study.

One of the most severe complications related to endotracheal intubation is laryngeal edema, which may present as upper airway obstruction that can lead to respiratory failure requiring reintubation. The incidence of post-extubation laryngeal edema reported in the last 25 years with conventional endotracheal tubes ranges from 5 to 54.4%, depending on the definitions and method of diagnosis, and the incidence of stridor varies widely, ranging from 1.6 to 26.3% [21]. Little information is available about the incidence of complications with the use of endotracheal tubes with CASS. In 2004, analyzing the utility of CASS and the semi-recumbent position as methods of VAP prevention, Girou et al. [10] found laryngeal edema in two out of five patients, suggesting that this method of prevention might be unsafe. However, it is important to note that the aspiration pressure applied in that study was 30 mmHg. Moreover, some recent clinical reports suggest that device malfunctioning may cause tracheal mucosa to be drawn into the hole of the aspiration channel, obstructing the system and subsequently injuring the mucosa [11, 12].

In our prospective study, the incidence of upper airway obstruction was 8.1%. Previous studies reported that the upper airway obstruction was more common in women and in patients with larger endotracheal tubes, longer mechanical ventilation and traumatic or difficult intubation [22, 23]. Our findings partially corroborate these results as we found that intubation-related factors associated with upper airway obstruction were the number of attempts required for intubation (*p* = 0.001), difficult intubation (*p* = 0.001) and a higher Cormack score (*p* = 0.003). The size of the endotracheal tube in our study was not identified as a risk factor for upper airway obstruction despite the fact that the accessory channel for subglottic aspiration increases the external diameter in these tubes.

Reintubation is necessary in 10–100% of patients with post-extubation laryngeal edema or stridor [21]. In our study, the rate of reintubation due to upper airway obstruction was 87.5%, representing a third of all patients who required reintubation. The mortality in patients who required reintubation was 33.3%, but the crude mortality in the group of patients with upper airway obstruction who required reintubation was 14.2% and suggests that, unlike reintubation for other reasons, reintubation for transitory upper airway obstruction is probably not associated with increased mortality.

Extubation failure occurs in about 10–20% of patients who meet weaning criteria and pass a spontaneous breathing trial [24]. In our study, the overall incidence of extubation failure requiring reintubation was 20.9%. However, the incidence in the group with accidental or self-extubation (42.8%) was higher than in the group with planned extubation (18.9%). In addition, it is important to highlight that a large proportion (46.6%) of the patients needing reintubation in the group with planned extubation had neurological disorders. In several studies, neurological disorders or impaired neurological status is independently associated with extubation failure [25–27].

About 10% of critically ill patients who require mechanical ventilation undergo tracheostomy [28–30]. The incidence of tracheostomy in our study was 12.7%. This incidence is similar to the 13% reported in a recent study in 31 ICUs throughout Spain [31].

Some limitations of our study should be taken into account: It was carried out in a single medical surgical ICU with extensive experience in the use of endotracheal tubes with CASS and aspiration pressure was strictly controlled. Caution is therefore warranted if our results are extrapolated to other contexts. A substantial limitation is that the study was observational and not randomized versus conventional endotracheal tubes. However, in our ICU all patients use CASS to prevent VAP, and we considered it unethical to increase the risk of pneumonia in a group without CASS. Another potential limitation is that MDCT might underestimate the incidence of structural lesions of the upper airway. However, MDCT’s sensitivity in the diagnosis of severe and significant tracheal lesions is higher than 90% [17–20]. Another limitation is due to the fact that MDCT was only performed in 43% of cases for safety reasons, and we cannot be sure that the incidence of lesions in the entire group was not higher. However, the absence of significant clinical manifestations makes this unlikely. And finally, the mean time from extubation to evaluation to tracheal lesions was 5 ± 2 days. Moderate lesions could have been missed, and thus, the incidence might have been underestimated.

## Conclusion

The risk of clinically significant complications such as extubation failure related to upper airway obstruction in patients with CASS was 8.1% and the subglottic and tracheal lesions observed by computed tomography were not severe and located mostly in subglottic space.

## Abbreviations

ICU: Intensive care unit
VAP: Ventilator-associated pneumonia
CASS: Continuous aspiration subglottic secretions
APACHE II: Acute Physiology and Chronic Health Evaluation II
COPD: Chronic obstructive pulmonary disease
MDCT: Multidetector computed tomography
